# Daily consumption of monosodium glutamate pronounced hypertension and altered renal excretory function in normotensive and hypertensive rats

**DOI:** 10.1016/j.heliyon.2022.e10972

**Published:** 2022-10-05

**Authors:** Nattaya Thongsepee, Pongsakorn Martviset, Pathanin Chantree, Phornphan Sornchuer, Kant Sangpairoj, Parisa Prathaphan, Jittiporn Ruangtong, Siriphun Hiranyachattada

**Affiliations:** a Department of Preclinical Science, Faculty of Medicine, Thammasat University, Pathum Thani, 12120, Thailand; b Research Unit in Nutraceuticals and Food Safety, Thammasat University, Pathum Thani, 12120, Thailand; c School of Pharmacy, Walailak University, Nakhon Si Thammarat, 80160, Thailand

**Keywords:** Monosodium glutamate, Hypertension, Renal function, 2K1C

## Abstract

This study aimed to investigate the effects of monosodium glutamate (MSG) on the levels of arterial blood pressure (ABP) and renal excretory function. Male Wistar rats were divided into 2 groups (n = 24 each) namely sham operation (SO) and 2-kidneys-1-clip (2K1C) to develop the normotensive and hypertensive model, respectively. Four weeks after the operation, each group of rats were further divided into 4 subgroups (n = 6 each) which were orally administered of either distilled water or MSG at the doses of 80, 160, or 320 mg/kg BW/day once a day for 8 weeks. The body weight, the 24-hour water intake, and the 24-hour urine output were recorded weekly. Then, each rat was anesthetized and the ABP was measured via carotid artery. The renal excretory function was examined by using the clearance technique to measure the levels of the glomerular filtration rate and the renal blood flow. The levels of serum malondialdehyde (MDA) as a marker of oxidative stress were analyzed. The expression of tumor necrosis factor alpha (TNF-α) in the kidneys was also investigated using immunohistochemistry. It was found that all doses of MSG significantly increased the ABP in both SO and 2K1C groups. All doses of MSG significantly increased the serum MDA levels and the expression of TNF-α in the kidneys of the SO groups. Long-term intake of 320 mg/kg BW MSG significantly decreased the renal excretion of salt and water in both SO and 2K1C groups. As a whole, this study demonstrated that MSG consumption contributed to an increase in oxidative stress which could lead to alterations in the cardiovascular and renal function.

## 1 Introduction

Monosodium glutamate (MSG, C_5_H_8_NNaO_4_.H_2_O) is a sodium salt of glutamic acid which has been used as the most famous flavor enhancer. MSG has a white, odorless crystalline powder characteristic and contains 78% glutamic acid, 21% sodium, 1% contaminant and water [1, 2]. The Food and Drug Administration considers that MSG is safe when used within a limited amount; however, the safe dose of MSG for long-term daily intake is not yet specified. Some Thai manufacturers recommended the dose of MSG for a dish meal on the packet is 1 teaspoon (4 g). Despite that, MSG has been used with an unlimited amount in restaurants, cafeterias, street foods, and packet foods. The study in a rural Thai population demonstrated an average MSG intake of 4.0 ± 2.2 (range 0.4–14.0) g/day, and every 1 g increase in MSG intake significantly increased the risk of metabolic syndrome or development of an overweight condition regardless of the total energy intake or the level of physical activity [3].

It has been reported that MSG administration affected various human physiologic functions. Intraperitoneal injection of MSG (4 g/kg BW) for 10 days markedly increased malondialdehyde (MDA) levels in the liver, kidney, and brain of the rats [4], and oral gavage of MSG (0.6 g/kg BW) for 10 days induced lipid peroxidation, decreased glutathione (GSH) level, and increased the activities of glutathione-s-transferase (GST), catalase (CAT), and superoxide dismutase (SOD) in the liver of the rats [5]. Furthermore, MSG has been shown to alter metabolic functions. Twice subcutaneously injection of MSG (4 g/kg BW) on the offspring rats following by feeding with hypercaloric diet for 45 days induced alterations in a metabolic rate of glucose utilization and decreased antioxidant defense [6]. Application of MSG in drinking water (2 g/g BW) to rats significantly lowered pancreatic β-cell mass at 1, 6, and 9 months after the treatment; however, serum insulin levels, glucose tolerance, body weight, and food consumption in MSG-treated and control rats were still similar. Interestingly, the water intake was significantly higher in the MSG-treated group at all time points [7]. In addition, MSG was used to induce the obesity in a rodent model; subcutaneous injection of MSG (2 mg/kg BW) in the first seven days of life markedly increased the BW, Lee index, and epididymal white adipose tissue of rats. This obesity induced by neonatal MSG treatment impaired cardiac autonomic function and most likely contributed to increased mean arterial pressure as well as insulin resistance [8]. MSG also showed a nephrotoxic effect in rats. Oral administration of MSG (4 g/kg BW) for 180 days had significantly increased renal function markers including serum urea, uric acid, and creatinine, lipid peroxidase byproducts (MDA and conjugated dienes), and decreased the levels of SOD, CAT, GST, GSH, and glutathione peroxidase (GPx) in the kidneys. Moreover, the kidneys in the MSG-treated group showed congested glomeruli, tubular swelling, capillary congestion, and microhemorrhages in stromal areas of the tubules [9]. However, the doses of MSG in the aforementioned studies did not show their relation to a habitually daily intake of an individual and the safe dose of MSG for long-term daily consumption has not been specified.

This study aimed to investigate the effects of 8 weeks daily intake of MSG on the level of arterial blood pressure (ABP) and renal excretory function in the 2-kidneys-1-clip (2K1C) hypertensive and the normotensive model. The 2K1C model involves unilateral stenosis of the renal artery leading to a permanent reduction in renal perfusion pressure in a clipped kidney. The induced hypertension is dependent upon activation of the renin-angiotensin-aldosterone system (RAAS), which plays an important role in the control of cardiovascular homeostasis affecting both ABP and body fluid volume. The applied doses of MSG were 80, 160, and 320 mg/kg BW which refer to daily intake of 1.5, 3, and 6 teaspoons, respectively, in human adult. The body weight of each animal was recorded weekly. The effects of MSG on the 24-hour water intake and the 24-hour urine output were investigated weekly by a metabolic cage study. The serum MDA level, a marker of lipid peroxidation, was measured using a thiobarbituric reaction (TBAR) assay. An expression of tumor necrosis factor alpha (TNF-α) in left and right kidneys of those rats were also examined using an immunohistochemistry.

## 2 Materials and methods

### 2.1 Animals

Male Wistar rats (5-week-old, n = 48) were purchased from the Siam Nomura International Co. Ltd. (Thailand). They were housed under standard control condition (temperature of 22 ± 1 °C, relative humidity 30–70%, light 130–325 Lux, 12/12 h dark/light cycle, noise <86 dB) at the Laboratory Animal Center, Thammasat University, Pathum Thani, Thailand. The rats were fed with standard chow diet and reverse osmosis water *ad libitum*. The rats were acclimatized for a week before starting the experiment. The experimental protocols were adhered to NIH Guiding Principles in the Care and Use of Animals and were approved by the Thammasat University Animal Care and Use Committee under Protocol No. 028/2019.

### 2.2 Hypertensive rat model induction

Rats were randomly divided into 2 groups (24 animals each) for undergoing either sham operation (SO) or 2K1C to develop the normotensive or the hypertensive model, respectively. Each rat was anaesthetized with 5% isoflurane (RWD, Shenzhen, China) supplied with oxygen (O_2_, 4 L/min) for one minute. Then, each rat was placed on an electrical temperature control pad and anesthesia maintained with 0.9–2% isoflurane and O_2_ (0.9 L/min) using a facemask throughout the operation. The left kidney was exposed through a 1-cm retroperitoneal incision. The left renal artery was cleared from surrounding fat and connective tissues, and then was clipped with a U-shaped silver clip with a 0.20 mm gap. The muscle and skin layer were sutured separately with 4/0 catgut. SO rats had similar entire surgery except for renal artery clipping. Rats were left to recover in separate cages for 30 min. Carprofen (5 mg/kg BW) was given subcutaneously for pain relief once daily for 3 days. All animals were convalesced over 4 weeks to allow the development of hypertension.

### 2.3 MSG administration

**Figure 1:**
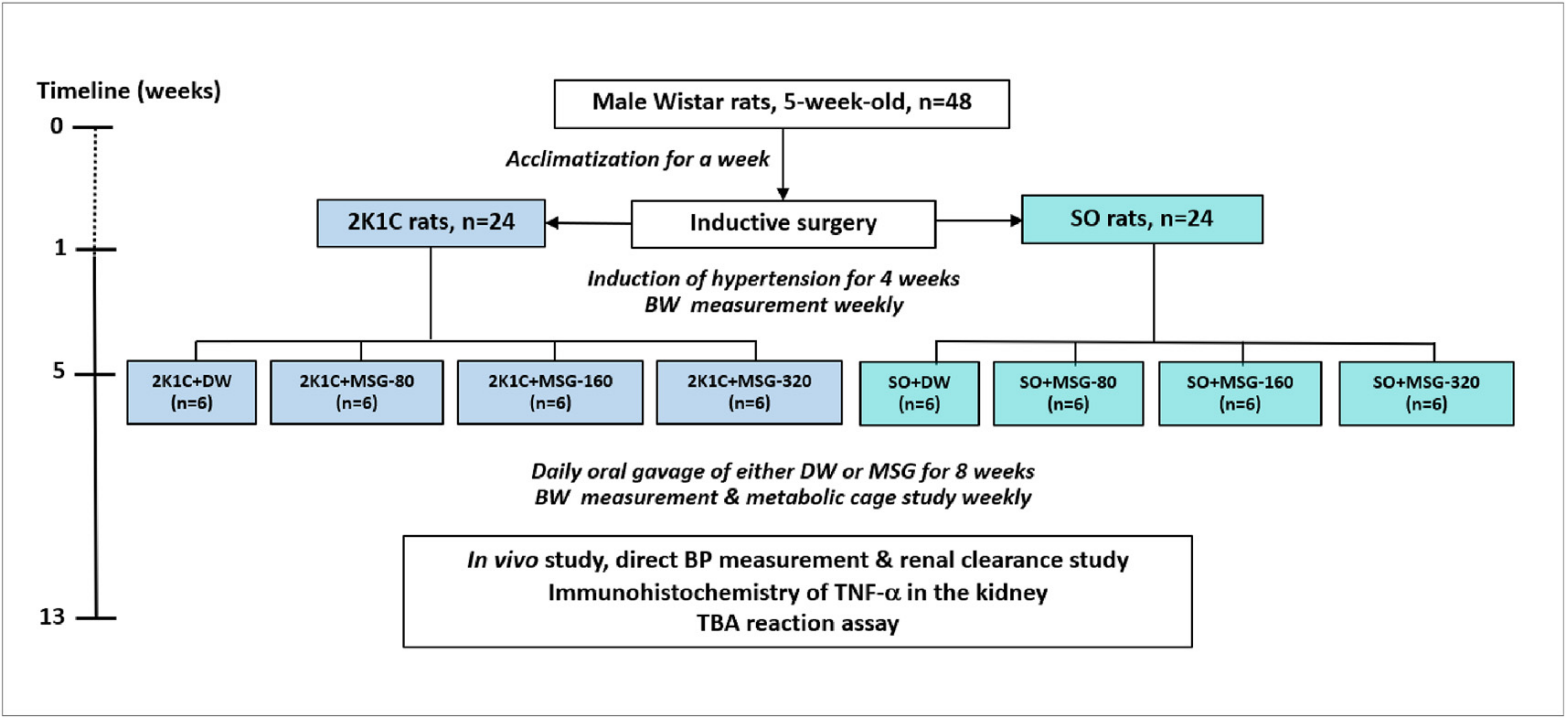
Schematic diagram of the experimental protocol. Abbreviation: 2-kidneys-1-clip, 2K1C; sham operation, SO; monosodium glutamate, MSG; blood pressure, BP; distilled water, DW; body weight, BW; tumor necrosis factor alpha, TNF-α; thiobarbituric acid, TBA.

After the inductive phase, each group of rats was further divided in to 4 subgroups (6 each) as following: Group I (control), the rats were given a distilled water, and Group II to IV, the rats were daily orally administered with MSG solution at the dose of 80, 160, and 320 mg/kg BW, respectively, for 8 weeks. The doses of MSG were calculated based on a recommended dose indicated on the commercial packet from Thai manufacturer for a dish meal which is 1 teaspoon (about 4 g). Generally, a human individual has 3 meals a day, so the amount of MSG consumption would be 12 g/day or about 150–170 mg/kg BW/day (based on an average 70–80 kg bodyweight of normal adults). In this study, the administered MSG were designed at the dose of 80, 160, and 320 mg/kg BW/day which equivalent to 1.5, 3, and 6 teaspoons of MSG consumed in human, respectively. The MSG was dissolved in distilled water and the volume of oral gavage was 2.5 mL/kg BW in all groups. The schematic diagram of the experimental protocol was represented in Figure 1.

### 2.4 Body weight, 24-hour water intake, and 24-hour urine output determination

The BW was recorded weekly throughout the study, and the BW change represented the difference between the BW before and 8 weeks after MSG consumption. The 24-hour water intake and the 24-hour urine output were recorded weekly after MSG administration using a metabolic cage.

### 2.5 ABP measurement and renal clearance study

After MSG administered phase, all rats were anesthetized again as previously described. The direct ABP measurement was performed via the left carotid artery cannulation using polyethylene (PE) tube containing a heparinized 0.9% NaCl connecting to the pressure transducer and the PowerLab system (model 26T, ADInstruments, New South Wales, Australia) for continuously ABP and heart rate (HR) recording throughout the experiment.

The renal excretory function was examined as following: the right jugular vein was cannulated with PE tube for infusion of the renal clearance markers including 1% para-amino hippuric acid (PAH) and 1% inulin (Sigma-Aldrich, Darmstadt, Germany) in 0.9% NaCl at a rate of 1.6 mL/min/100 g BW. The urinary bladder was also cannulated with the PE tube for urine sampling. After infusion of clearance marker solution for 30 min, urine samples were collected every 30 min in the pre-weighed tube for 4 periods which were called U1 – U4. Arterial blood (1 mL) was collected at the end of U1 and U3 via the carotid artery in the heparinized tubes. A small amount of blood was used to measure the hematocrit, and the remaining sample was centrifuged at 4,000 RPM for 10 min for plasma collection. The plasma and urine samples were kept at -20 °C for analysis of the PAH and inulin levels using spectrophotometry following the methods of Smith et al. [10] and Davidson and Sackner [11], respectively. The plasma (P_osm_) and urine osmolarity (U_osm_) were measured using a Micro-Osmometer (model Osmomat 030D, Gonotec, Berlin, Germany).

Mass blood collection was performed at the end of experimentation, the blood sample was centrifuged at 4,000 RPM for 10 min, and the serum was kept at -40 °C. The weight of kidneys, heart, and liver were recorded after sacrifice the rat by perfusion of phosphate buffer solution (PBS). The left and right kidneys were fixed in 4% paraformaldehyde for histological study.

### 2.6 TBAR assay

The level of serum MDA was used as a marker of lipid peroxidation and oxidative stress. The MDA levels were measured in the form of TBA reacting substances. All reagents were purchased from Sigma-Aldrich (Darmstadt, Germany). The protocol was modified from Costa et al [12]. Briefly, 100 μL of serum was mixed with 1 mL of a cocktail solution containing 15% trichloroacetic acid, 0.38% TBA, and 0.25 M hydrochloric acid (HCl). The mixture was heated at 95 °C for 30 min and then was cooled on ice for 2 min. Then, centrifugation at 10,000 g for 5 min and the absorbance of the supernatant was measured at 532 nm. For the MDA standard preparation, a stock of solution of 100 μL of MDA was prepared in 0.01 mmol/L HCl and dilutions from stock solution of 20–0.25 μmol/L were performed.

### 2.7 Immunohistochemistry of TNF-α in the kidneys

The fixed left and right kidneys were processed and embedded in paraffin. The tissues were cut at 5 μm thickness and mounted on the glass slides. The sections were deparaffinized, rehydrated, and antigen unmasked with 10 mM sodium citrate (pH 6.0) using microwave technique. The sections were blocked with 10% normal horse serum (ab7484, Abcam) in PBS for 1 h and then incubated overnight with an anti-TNF alpha antibody (mouse monoclonal, ab220210, 1:200, Abcam). After washing, the sections were incubated with 0.3% H_2_O_2_ for 15 min and with a goat anti-mouse IgG (H + L) cross-adsorbed secondary antibody, biotin (1: 5,000, Invitrogen) for 1 h. For detection, the sections were washed and further incubated with ABC peroxidase standard staining kit (Thermo Scientific™) for 30 min and with a DAB substrate kit (ab64238, Abcam) for 5 min. The sections were counter stained with hematoxylin solution modified acc. to Gill III (Sigma-Aldrich) for a few seconds. All slides were dehydrated and mounted with mounting medium. The images were capture in an imaging system (Nikon, Tokyo, Japan).

### 2.8 Calculation

The parameters of ABP (mm Hg) compose of systolic blood pressure (SBP), diastolic blood pressure (DBP), and pulse pressure (PP) were analyzed at the mid-period of U1 – U4. The mean arterial blood pressure (MAP) was calculated from standard equation, MAP = DBP +1/3(SBP - DBP). The renal vascular resistance [RVR, resistance unit (RU)] were calculated using the formula, RVR = MAP ÷ renal blood flow (RBF). All values from U1 to U4 periods of each rat were averaged.

The urine flow rate $[\dot{\text{V}}$, μL/min/g kidney weight (KW)] was computed by assuming a density of 1 g/mL. The inulin clearance (C_in_, refer to GFR, mL/min/g KW), PAH clearance (C_PAH_, refer to an effective renal plasma flow (ERPF), mL/min/g KW), and osmolar clearance (C_osm_, μL/min/g KW) were calculated from standard renal clearance equation [C_x_ = (U_x_ · $\dot{\text{V}}$) ÷ P_x_], when U_X_ = concentration of X in urine (mg/mL or mOsm/kg H_2_O), P_X_ = concentration of X in plasma (mg/mL or mOsm/kg H_2_O). The ERPF was converted to renal plasma flow (RPF = ERPF ÷ 0.9) and RBF [RBF = (RPF ÷ (1 – hematocrit)], respectively. Free water clearance (CH_2_O, μL/min/g KW) was computed according to the formula: CH_2_O = $\;\dot{\text{V}}$ – C_osm_. The values of $\dot{\text{V}}$, C_in_, C_PAH_, C_osm_, and CH_2_O were normalized by kidney weight and the data from U1 to U 4 of each rat were averaged.

### 2.9 Statistical analysis

Data were presented as mean ± standard error of the mean (S.E.M.). Comparisons between the means values within-group and among groups of the SO and the 2K1C were performed with two-way analysis of variance (ANOVA) followed by the multiple *t*-tests using GraphPad Prism 8 (San Diego, CA, USA). A *p*-value of less than 0.05 was considered a significant difference.

## 3 Results

### 3.1 The serum MDA levels

**Figure 2:**
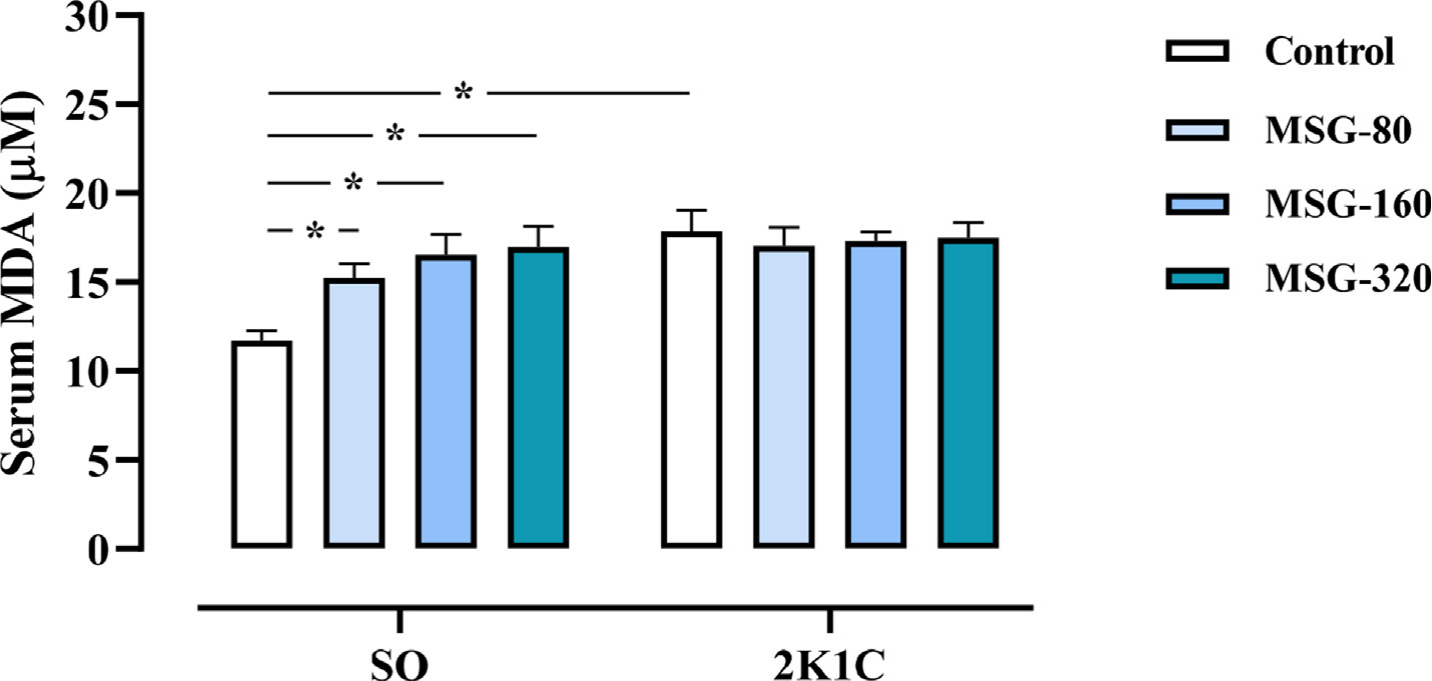
Effects of daily consumption of monosodium glutamate (MSG) for 8 weeks at the dose of 80, 160, and 320 mg/kg BW/day on the serum malondialdehyde (MDA) levels in the sham operative (SO) and the 2-kidneys-1-clip (2K1C) groups. Data expressed as mean ± S.E.M. ∗p < 0.05 in comparison between groups.

The levels of serum MDA in all groups of 2K1C rats were significantly higher than the values in the SO control (SO control: 11.80 ± 0.56; 2K1C control: 17.85 ± 1.30; 2K1C + MSG-80: 17.16 ± 0.95; 2K1C + MSG-160: 17.31 ± 0.55; 2K1C + MSG-320: 17.48 ± 0.96 μM/mL, *p* < 0.05). Within the SO groups, the levels of serum MDA in all MSG-treated groups were significantly increased in comparison to the control (SO + MSG-80: 15.24 ± 0.87; SO + MSG-160: 16.54 ± 1.25; SO + MSG-320: 16.91 ± 1.14 μM/mL, *p* < 0.05, Figure 2). The serum MDA levels did not different among the groups of 2K1C.

### 3.2 The weights of body, kidney, and heart

There was no significant difference in the body weights among all groups. The relative weights of right kidney and heart in all groups of 2K1C were significantly increased while left kidney weights were significant decreased in comparison to the respective values of SO control group (Table 1).

**Table 1:** Weights of body (BW), left kidney, right kidney, and heart of the 2-kidneys-1-clip (2K1C) hypertensive and the sham operation (SO) groups after daily administration of either distilled water (control) or monosodium glutamate (MSG) at the dose of 80, 160, and 320 mg/kg BW/day for 8 weeks.

Body/organ weight	SO				2K1C			
	Control	MSG-80	MSG-160	MSG-320	Control	MSG-80	MSG-160	MSG-320
BW changes (g)	177 ± 6	196 ± 10	201 ± 11	182 ± 4	188 ± 14	201 ± 11	203 ± 11	200 ± 6
Left kidney (g% BW)	0.30 ± 0.01	0.30 ± 0.01	0.30 ± 0.02	0.29 ± 0.01	0.27 ± 0.01∗	0.26 ± 0.01∗	0.25 ± 0.01∗	0.26 ± 0.01∗
Right kidney (g% BW)	0.29 ± 0.01	0.30 ± 0.01	0.29 ± 0.01	0.28 ± 0.01	0.32 ± 0.01∗	0.32 ± 0.01∗	0.32 ± 0.01∗	0.32 ± 0.01∗
Cardiac mass (g% BW)	0.23 ± 0.01	0.24 ± 0.01	0.24 ± 0.01	0.24 ± 0.01	0.26 ± 0.01∗	0.27 ± 0.01∗	0.27 ± 0.01∗	0.26 ± 0.01∗

BW change = BW after MSG administration − BW before MSG administration.

The weight of each organ was represented as the relative organ weight (g% BW).

Data expressed as mean ± S.E.M. ∗p < 0.05 in comparison to SO control.

### 3.3 The water intake and the urine output in 24 h

**Figure 3:**
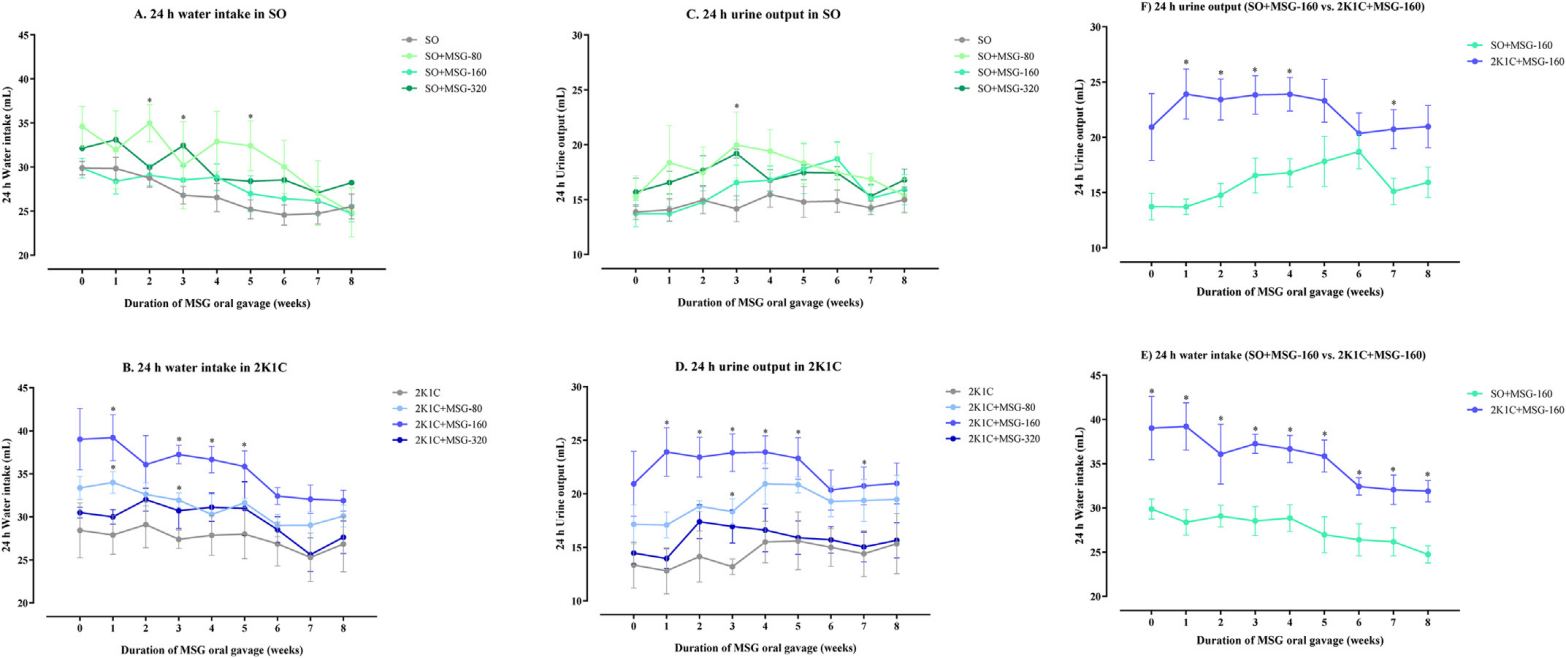
Effects of daily consumption of monosodium glutamate (MSG) for 8 weeks at the dose of 80, 160, and 320 mg/kg BW/day on the levels of the 24-hour water intake in the sham operative (SO, A) and the 2-kidneys-1-clip groups (2K1C, B) and the 24-hour urine output in the SO (C) and the 2K1C (D) groups along 8 weeks of administration. The effect of 160 mg/kg BW/day MSG on 24-hour water intake (E) and 24-hour urine output (F) in 2K1C compared to the SO groups along 8 weeks of oral administration. Data expressed as mean ± S.E.M. ∗p < 0.05 in comparison between groups.

In both SO and 2K1C groups, the levels of the 24-hour water intake and the 24-hour urine output were significantly increased in the early phase (1^st^ – 5^th^ week) of MSG administration compared with the respective control groups; however, those differences were absent in the late phase (6^th^ – 8^th^ week, Figure 3A – 3D). At the dose of 160 mg/kg BW of MSG, the levels of the 24-hour water intake in the 2K1C group were significantly higher than those of the SO group along 8^th^ weeks of administration (Figure 3E) and the levels of the 24-hour urine output in the group of 2K1C were significantly higher than those of the SO group during the early phase of administration (Figure 3F).

### 3.4 The arterial blood pressure

**Figure 4:**
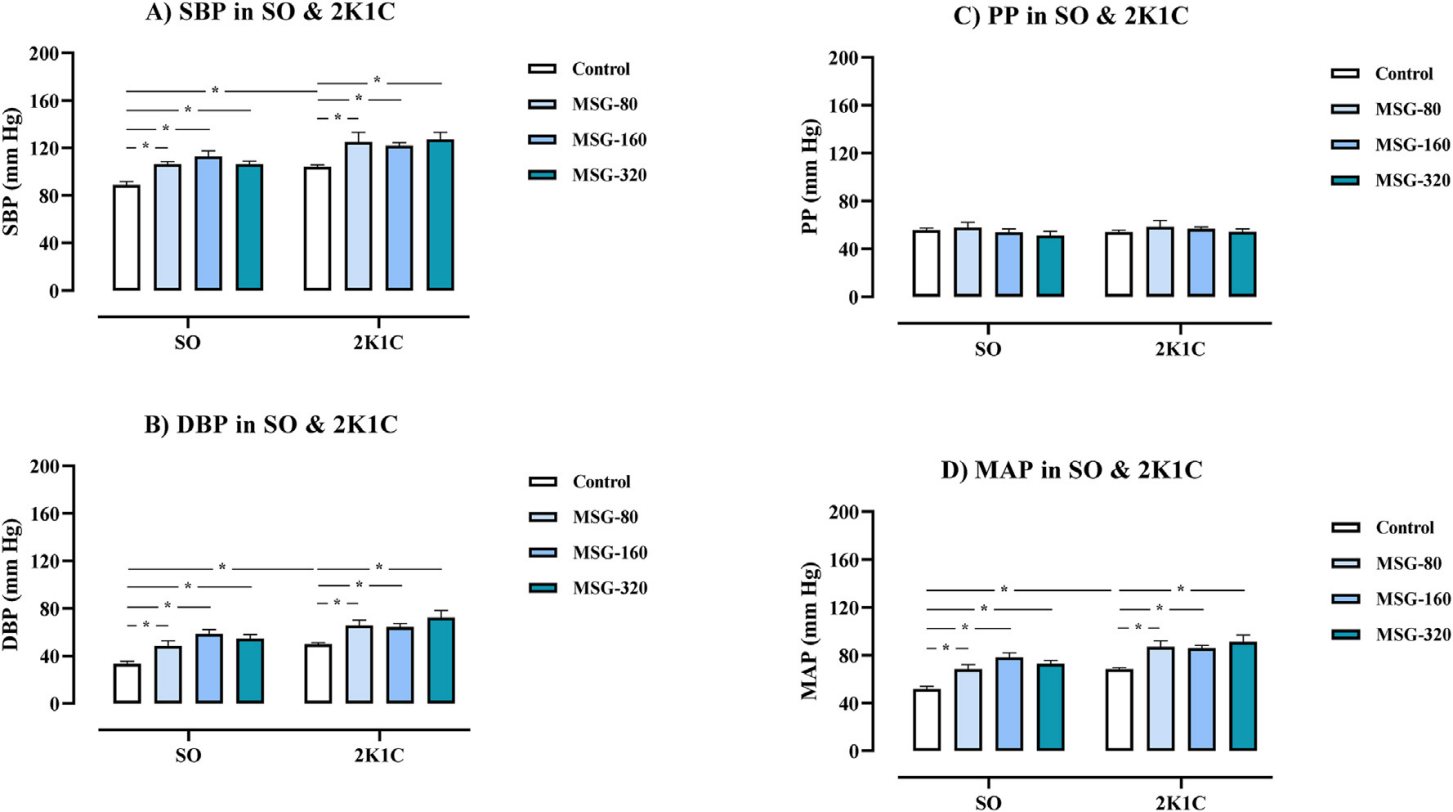
Effects of daily consumption of monosodium glutamate (MSG) for 8 weeks at the dose of 80, 160, and 320 mg/kg BW/day on systolic blood pressure (SBP, A), diastolic blood pressure (DBP, B), pulse pressure (PP, C), and mean arterial blood pressure (MAP, D) in the sham operative (SO) and the 2-kidneys-1-clip (2K1C) groups. Data expressed as mean ± S.E.M. ∗p < 0.05 in comparison between groups.

The levels of SBP, DBP, and MAP in all groups of 2K1C rats were significantly higher than the values in the SO control (SO control: SBP = 89 ± 3, DBP = 33 ± 2, MAP = 52 ± 3; 2K1C control: SBP = 104 ± 2, DBP = 50 ± 1, MAP = 68 ± 1; 2K1C + MSG-80: SBP = 125 ± 9, DBP = 66 ± 5, MAP = 87 ± 5; 2K1C + MSG-160: SBP = 122 ± 3, DBP = 64 ± 3, MAP = 86 ± 3; 2K1C + MSG-320: SBP = 127 ± 6, DBP = 72 ± 7, MAP = 91 ± 6 mm Hg, p < 0.05, Figure 4). Within the SO groups, the levels of SBP, DBP, and MAP in all groups of MSG significantly increased compared to respective values of the control (SO + MSG-80: SBP = 106 ± 2, DBP = 48 ± 5, MAP = 69 ± 4; SO + MSG-160: SBP = 113 ± 5, DBP = 59 ± 4, MAP = 78 ± 4; SO + MSG-320: SBP = 106 ± 3, DBP = 55 ± 4, MAP = 73 ± 3 mm Hg, p < 0.05). In the 2K1C rats, the levels of SBP, DBP, and MAP were also significantly increased compared to respective values of the control. There was no change in the PP and the HR among all groups.

### 3.5 The renal excretory function

**Figure 5:**
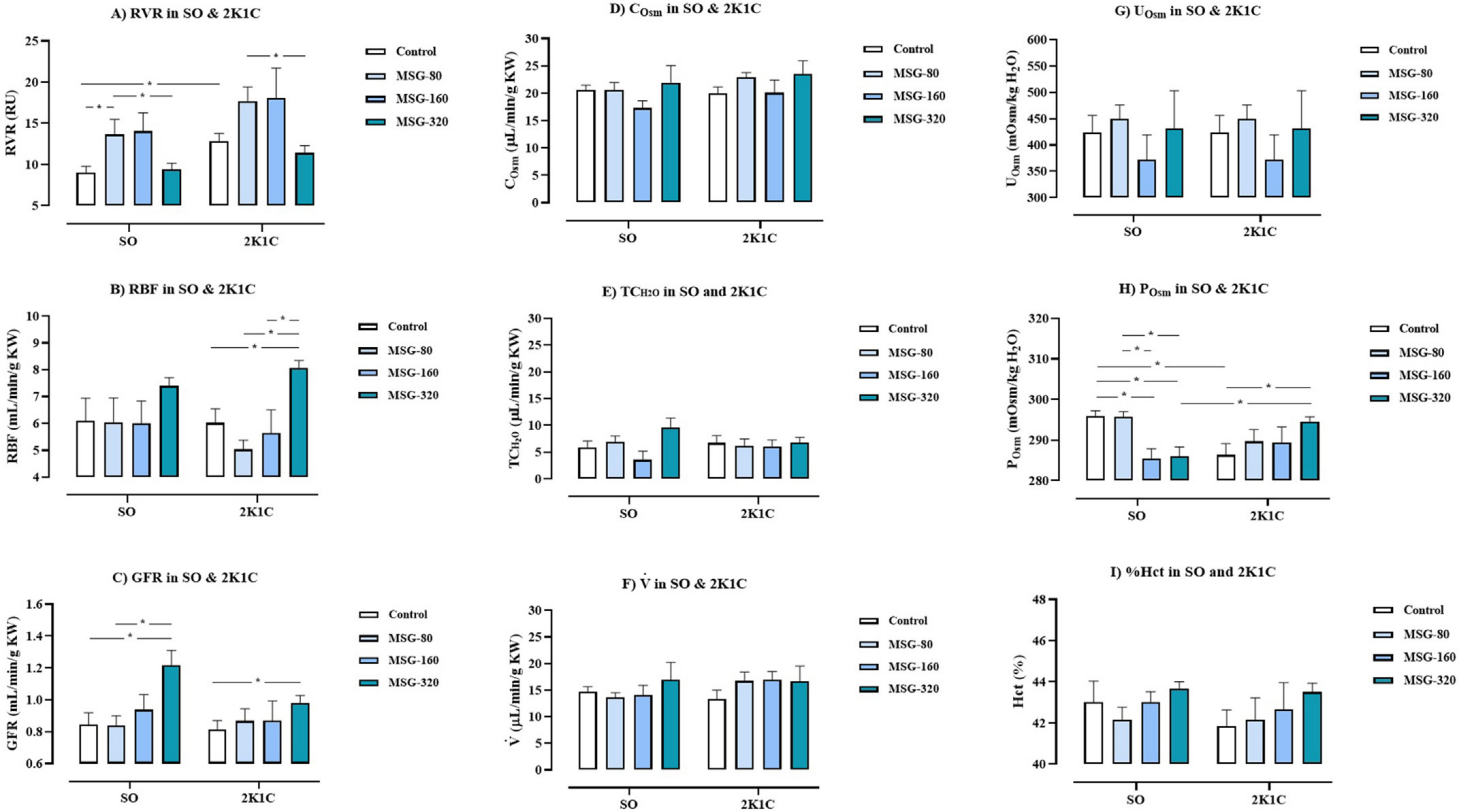
Effects of daily consumption of monosodium glutamate (MSG) for 8 weeks at the dose of 80, 160, and 320 mg/kg BW/day on renal vascular resistance (RVR, A), renal blood flow (RBF, B), glomerular filtration rate (GFR, C), osmolar clearance (C_osm_, D), negative free water clearance (TCH_2_O, E), urine flow rate ($\dot{\text{V}}$, F), urine osmolarity (U_osm_, G), plasma osmolarity (P_osm_, H), and hematocrit (I) in the sham operative (SO) and the 2-kidneys-1-clip (2K1C) groups. Data expressed as mean ± S.E.M. ∗p < 0.05 in comparison between groups.

As shown in Figure 5A, the levels of RVR in the groups of 2K1C control, 2K1C + MSG-80, and 2K1C + MSG-160 but not 2K1C + MSG-320 were significantly higher than the values of SO control (SO control: 8.99 ± 0.85, 2K1C control: 17.06 ± 3.19, 2K1C + MSG-80: 17.71 ± 1.86, 2K1C + MSG-160: 18.07 ± 3.98, 2K1C + MSG-320: 11.42 ± 0.11 RU, p < 0.05). Within the SO groups, the levels of RVR in the SO + MSG-80 group were significantly higher than the control and the SO + MSG-320 groups (SO + MSG-80: 13.86 ± 1.65, SO + MSG-160: 14.05 ± 2.43, SO + MSG-320: 9.41 ± 0.80 RU, p < 0.05). Within the 2K1C groups, the levels of RVR in the MSG-80 group were significantly higher than the values in the MSG-320 group.

The levels of RBF between the SO and the 2K1C groups were not different (Figure 5B). There was no different in the levels of RBF among the groups of SO. Within the 2K1C groups, the levels of RBF in the MSG-320 group were significantly higher than value of the control, the MSG-80, and the MSG-160 groups (2K1C control; 6.02 ± 0.57, 2K1C + MSG-80: 5.03 ± 0.38, 2K1C + MSG-160: 5.64 ± 0.94, 2K1C + MSG-320: 8.07 ± 0.30 mL/min/g KW, p < 0.05).

The levels of GFR between the SO and the 2K1C groups were not different as shown in Figure 5C. Within the SO groups, the levels of GFR in the MSG-320 group were significantly higher than those of the control and the MSG-80 groups (SO control: 0.84 ± 0.08, SO + MSG-80: 0.84 ± 0.07, SO + MSG-160: 0.94 ± 0.10, SO + MSG-320: 1.22 ± 0.10 mL/min/g KW, p < 0.05). Within the 2K1C groups, the levels of GFR in the MSG-320 group were significantly increased compared with the control (2K1C control: 0.81 ± 0.06, 2K1C + MSG-80: 0.87 ± 0.08, 2K1C + MSG-160: 0.87 ± 0.14, 2K1C + MSG-320: 0.98 ± 0.05 mL/min/g KW, p < 0.05).

The levels of P_osm_ in the 2K1C control were significantly lower than the values in SO control (Figure 5H). Within the SO groups, the levels of P_osm_ in the MSG-160 and MSG-320 groups were significantly lower than the control and the MSG-80 group (SO control: 296 ± 2, SO + MSG-80: 295 ± 1, SO + MSG-160: 286 ± 3, SO + MSG-320: 286 ± 2 mOsm/kg H_2_O, p < 0.05). The levels of P_osm_ in the 2K1C + MSG-320 group were significantly higher than the 2K1C control and the SO + MSG-320 group (2K1C control: 286 ± 3, 2K1C + MSG-80: 290 ± 3, 2K1C + MSG-160: 289 ± 4, 2K1C + MSG-320: 294 ± 1 mOsm/kg H_2_O, p < 0.05).

As shown in Figures 5D – 5G and 4I, the levels of C_osm_, CH_2_O, $\dot{\text{V}}$ , U_osm_, and hematocrit were not different among the groups of SO and 2K1C.

### 3.6 The renal TNF-α expression

**Figure 6:**
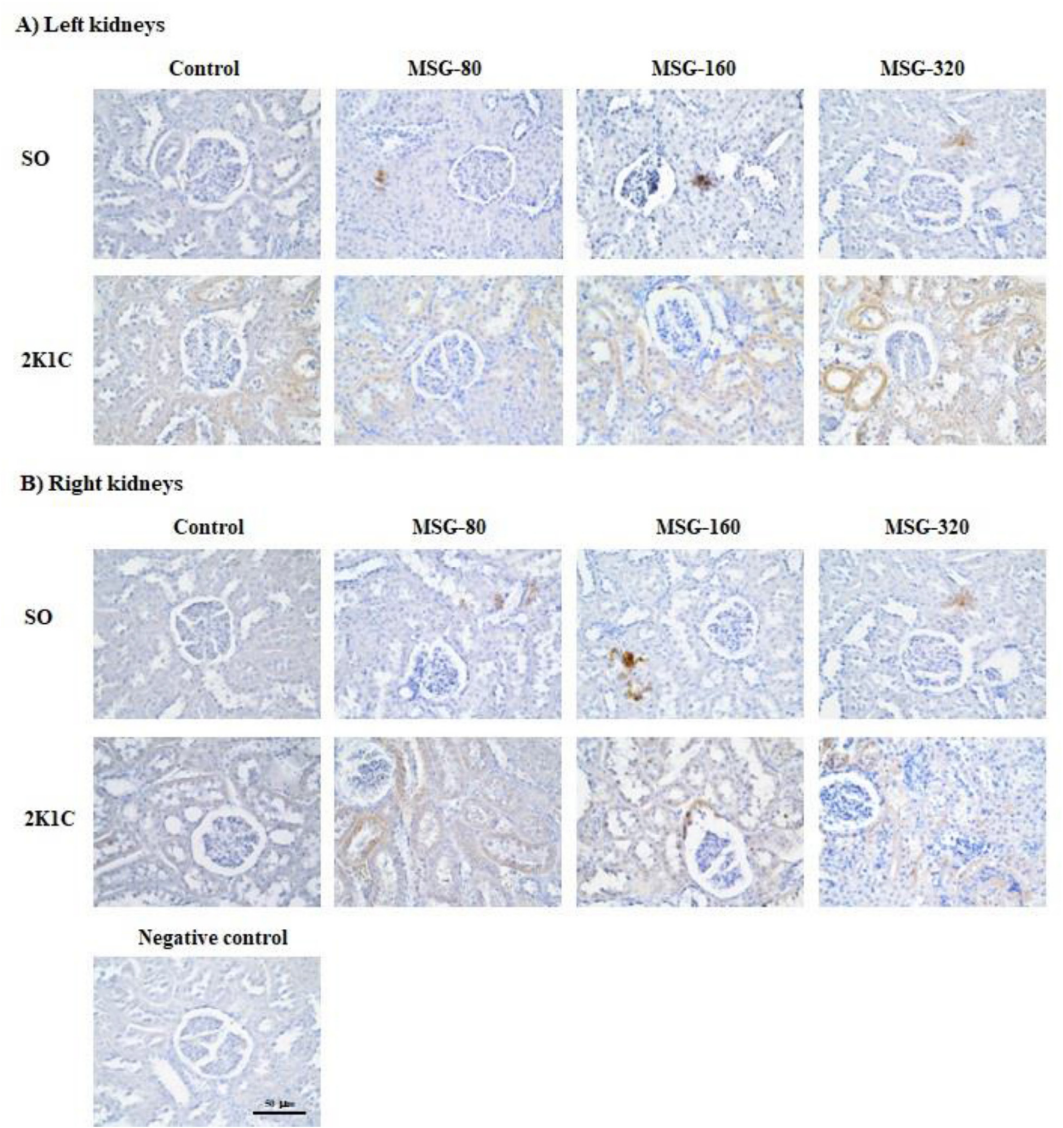
Immunohistochemistry of tumor necrosis factor alpha (TNF-α) in A) the left and B) the right kidneys of the sham operative (SO) and the 2-kidneys-1-clip (2K1C) rats after daily consumption of monosodium glutamate (MSG) at the dose of 80, 160, and 320 mg/kg BW/day for 8 weeks.

In SO groups, the expression of TNF-α in both left and right kidneys was increased in all groups of MSG in comparison to the control, however, it seemed there was no difference among the MSG-treated groups. In 2K1C groups, the TNF-α expression in both left and right kidneys was obviously more than the SO groups but it was similar among all groups of 2K1C (Figure 6).

## 4 Discussion

The induction of the hypertensive model was considered successful since the levels of ABP in the 2K1C control group were significantly higher than the SO control even in the anesthetized condition. The weights of right non-clipped kidneys of the 2K1C rats were significantly increased by 11.49% but the left clipped kidneys weights were significantly decreased by 7.91% compared with the SO control. However, the RBF and GFR in the 2K1C rats were comparable to the SO control rats suggesting that non-clipped kidney hypertrophy developed a mechanism of compensatory response to preserve the renal excretory function. Unlike the clipped kidneys, a decline of renal perfusion could stimulate the juxtaglomerular cells to release renin which further activated the RAAS. Renin converted angiotensinogen to angiotensin I which was subsequently converted to angiotensin II by an angiotensin converting enzyme. In this study, the levels of RVR in the 2K1C control group were significantly higher than the SO control suggesting a renal vasoconstriction induced by angiotensin II. Moreover, angiotensin II is likely to induce vasoconstriction throughout the body, resulting in an increased total peripheral resistance (TPR) and consequently increased afterload of the heart. It was found that the heart weight of the 2K1C control group were significantly increased in comparison to the SO control. This result suggests that hypertrophy of the heart may be due to an increase in either TPR or a direct hypertrophic effect of angiotensin II or both. Furthermore, angiotensin II could induce a release of aldosterone from the adrenal cortex, and as a result, an increase in the reabsorption of Na^+^ and water at the renal tubule. The finding showed that the P_osm_ levels in the 2K1C groups were significantly lower than the SO control suggesting the water expansion in the hypertensive groups. It is noted that the serum MDA levels and the expression of TNF-α in both clipped and non-clipped kidneys in 2K1C control was significantly higher than those of the SO control possibly due to the oxidative stress which was commonly occurred in the renovascular hypertensive model [13]. Overall, it was likely that an increase of ABP levels in the 2K1C groups was contributed by the oxidative stress, the vasoconstriction, and the retention of salt and water, in line with previous studies [14, 15, 16].

Daily consumption of MSG for 8 weeks induced oxidative stress. Among the SO groups, the levels of serum MDA in the groups of 80, 160, and 320 mg/kg BW MSG were significantly increased compared with the SO control in dose-dependent manner suggesting these doses of MSG may induce mild, moderate, and severe oxidative stress in those rats, respectively. The immunohistology shown that the expression of TNF-α in the kidneys of MSG groups were more marked than the control. This finding is consistent with previous studies: Farombi and Onyema reported that intraperitoneal injection of MSG 4 g/kg BW/day for 10 days significantly increased the levels of MDA, GST, CAT, and SOD but significantly decreased GSH levels in liver, kidneys, and brain [4], and Onyema et al. reported that oral gavage of MSG solution at the dose of 0.6 g/kg/day for 10 days significantly increased the levels of lipid peroxidation, GST, CAT, and SOD but decreased GSH level in the liver [5]. The mechanism of MSG-induced oxidative stress may involve a binding of glutamate with (N-methyl-D-aspartate) NMDA receptor. This causes an influx of Ca^2+^ which subsequently activates a protein kinase, a phospholipase, or nitric oxide synthase inside the cell, resulting in a generation of reactive oxygen species (ROS) and ultimately leading to a cell injury [17]. However, there was no different in the serum MDA levels among the groups of 2K1C because those values were sustained in the high levels due to the basal oxidative stress in the 2K1C model.

The long-term intake of MSG raised the ABP levels in the normotensive rats and increased the degree of hypertension in the hypertensive rats. The findings showed that all doses of MSG significantly increased the levels of SBP, DBP, and MAP of both SO and 2K1C groups in comparison to the respective control groups. Among the SO groups, the MAP in the MSG-80 and MSG-160 groups were significantly increased compared to the control in dose-dependent manner but it was slightly decreased in the MSG-320 group. It was interesting that there was some renal compensatory mechanism to limit the increase of ABP at the high dose of MSG intake. This finding is consistent with a previous study which reported that additional of MSG in the standard diet and drinking water of rats significantly increased the ABP levels, and its underlying mechanisms involving stimulation of oxidative stress as well as retention of Na^+^, K^+^ and water in the body [18].

Consumption of high dose of MSG for a long period affected the renal excretory function. At low dose, the oral gavage of 80 mg/kg BW/day MSG significantly increased the levels of RVR in the SO group, and its physiologic mechanism was likely associated with the oxidative stress that could induce the vascular endothelial dysfunction. The previous study reported that intraperitoneal injection of 4 g/kg BW/day MSG for 14 days to the newborn rats caused a vascular endothelial dysfunction [19]. Another study proposed the mechanism of action could involve the renal autoregulation which composes of the tubuloglomerular feedback (TGF) control and myogenic response. The TGF is the mechanism of macula densa at the early distal tubule in responses to an increased flow and NaCl concentration in tubular fluid by releasing a vasoconstrictor to the afferent arteriole in order to limit the RBF. The myogenic response is an activation of calcium channel at the vascular smooth muscle cell by high blood flow to induce vasoconstriction. At high dose, the present study demonstrated that oral administration of 320 mg/kg BW/day MSG significantly decreased the RVR levels parallel with significantly increased the levels of RBF and GFR in the SO and the 2K1C groups, suggesting an attribution of the vasodilation of renal vascular. Moreover, this finding suggested that the renal autoregulation in maintenance of GFR and RBF was impaired after long-term and high dose intake of MSG. These findings concurred with the previous study which reported that adding of 3 g/kg BW/day MSG to a standard diet 5 days a week, and spontaneous ingestion of a 1% MSG solution for 16 weeks significantly increased the RBF and GFR levels of rats in comparison to the relative dose of NaCl-treated and distilled water (DW)-treated rats [18]. Besides, it has been reported that the levels of urinary Na^+^ excretion were significantly increased in the MSG-treated and the NaCl-treated groups compared with the DW-treated group, but the urinary Na^+^ excretion levels of the MSG-treated group were significantly lower than the NaCl-treated group. Interestingly, the levels of urinary K^+^ excretion were significantly decreased in the MSG-treated group but were significantly increased in the NaCl-treated group in comparison to the DW-treated group. The urinary K^+^/Na^+^ ratio of the MSG-treated group was significantly lower than the NaCl-treated group suggesting that the MSG caused the K^+^ and Na^+^ retention more than the NaCl-treated group [18]. The vasodilation in the kidneys may result from the action of glutamate action that binds with the NMDA receptor [20]. In addition, the condition of K^+^ retention may induce hyperpolarization of the vascular smooth muscle cell leading to the vasodilation and increased blood flow [21]. Indeed, it was found that the ABP level was slightly decreased in the MSG-320 group in comparison to the MSG-160 group of SO rats, however, it was stilled significantly higher than that of the SO control. This finding suggested that the vasodilation in response to high dose of MSG was not strong enough for normalized the raised ABP. Further study is required to clarify the molecular mechanism about this response.

Long-term MSG administration altered the regulation of water balance in the body. This study found that MSG administration significantly increased the 24-hour water intake and the 24-hour urine output of the SO and 2K1C groups compared to the respective control groups in the early period (1^st^ – 5^th^ week). However, there was no different in the 24-hour water intake and the 24-hour urine output among the groups of the SO and 2K1C in late phase (6^th^ – 8^th^ week). These findings indicated that MSG stimulated thirst mechanism and increased urinary excretion in early phase of administration, but the regulation of the water balance may be disturbed in long-term. Among the SO groups, it was found that the P_osm_ levels in the groups of MSG-160 and MSG-320 were significantly lower than the control and MSG-80 groups suggesting that high doses of MSG caused a fluid overload in the body. However, within the 2K1C groups, the levels of P_osm_ in the MSG-320 were significantly higher than the control group suggesting that high dose of MSG decreased urinary salt excretion in this model of hypertension. When compared between the groups of SO and 2K1C which received the MSG at the dose of 160 mg/kg BW, it was found that the levels of 24-hour water intake in the 2K1C group were significantly higher than the values of the SO group throughout 8 weeks of MSG administration while the 24-hour urine output levels were significantly higher only in the early period of MSG administration. Besides, the P_osm_ levels of the SO group were significantly decreased, but the P_osm_ levels of 2K1C group did not change in comparison to the respective control. This finding was likely resulting from the severity of salt and water retention in the 2K1C group which was higher than the SO group because of the synergetic effects of RAAS and MSG in a reduction of salt and water excretion by the kidneys in the 2K1C group. Therefore, our findings suggest that MGS at the dose of 160 mg/kg BW could induce the salt and water retention easier in the hypertensive individual than in healthy one. The immunohistology shown that the TNF-α expression in all MSG groups were higher that the control suggesting the inflammation of renal tubular cell. Previous histological studies have showed that long-term administration of MSG caused the tubule-interstitial fibrosis, the glomerular hypercellularity, the tubular swelling, and the infiltration of inflammatory cells in the kidneys of rats [9, 22]. Therefore, high dose and long-term consumption of MSG may alter the structures and the excretory function of kidneys causing an alteration of the regulation of water balance of the body and ultimately leading to the hypertension.

Administration of MSG did not change the body weight of all groups of rats. This data was consistent with a previous study which reported that application of 1% MSG as drinking water to C57BL6/J mice treated with either low or high fat diet did not affect the body weight change of these mice [23].

**Figure 7:**
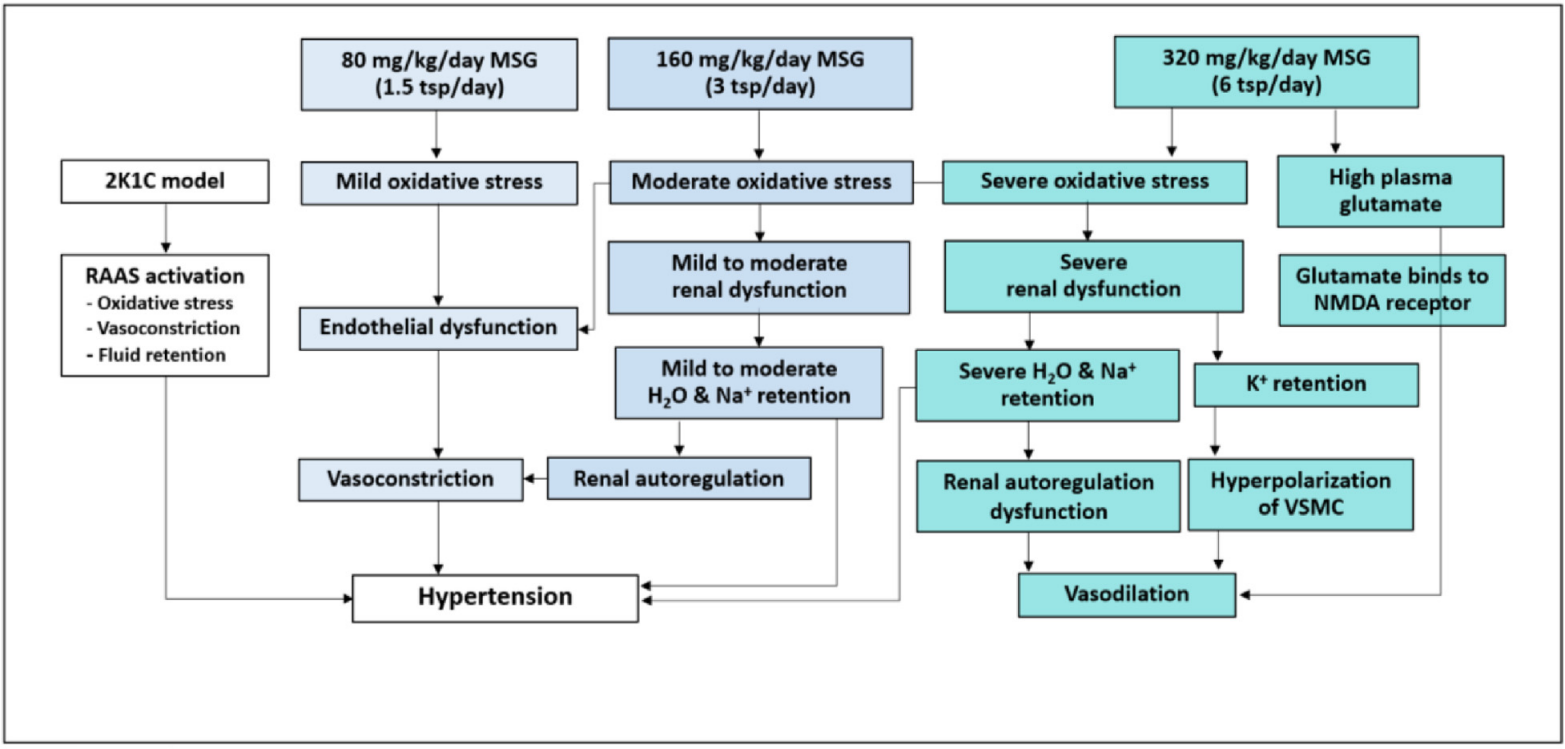
Schematic diagram of the mechanism of action of monosodium glutamate (MSG) induced hypertension and renal excretory dysfunction. Abbreviation: 2-kidneys-1-clip, 2K1C; renin-angiotensin-aldosterone system, RAAS; vascular smooth muscle cell, VSMC; anti-N-methyl D-aspartate, NMDA; teaspoon, tsp.

In conclusion (Figure 7), daily consumption of MSG at the dose of 80, 160, and 320 mg/kg BW/day which equivalent to 1.5, 3, and 6 teaspoons for adult, respectively, for 8 weeks induced the oxidative stress, increased ABP levels, and aggravated hypertension, however, the mechanism of action of each dose may different in some extension. Low dose of MSG (80 mg/kg BW/day) induced the oxidative stress and increased ABP levels in normotension and also aggravated a degree of hypertension in the patient. At moderate dose of MSG (160 mg/kg BW/day), it was not only increased the ABP but also affected the renal function by alteration of water balance and induction of salt and water retention which led to the hypertension. At high dose of MSG (320 mg/kg BW/day), the MSG could induce severe alteration of renal and vascular functions. The recommended dose for daily consumption of MSG is less than 6 g/day (1.5 teaspoons/day) for adult. The underlying mechanisms of MSG in alteration of water and electrolyte balance including an expression of NMDA receptor, aquaporin, K^+^ channel, and Na^+^ channel in the kidney require the further study.

## Declarations

### Author contribution statement

Nattaya Thongsepee: Conceived and designed the experiments; Performed the experiments Analyzed and interpreted the data; Contributed reagents, materials, analysis tools or data; Wrote the paper.

Pongsakorn Martviset, Pathanin Chantree, Phornphan Sornchuer, Kant Sangpairoj, Parisa Prathaphan, and Jittiporn Ruangtong: Performed the experiments; Analyzed and interpreted the data; Contributed reagents, materials, analysis tools or data; Wrote the paper.

Siriphun Hiranyachattada: Analyzed and interpreted the data; Wrote the paper.

### Funding statement

Dr. Nattaya Thongsepee was supported by Faculty of Medicine, Thammasat University, Thailand [Grant no. 2–15/2564] and and Research Unit in Nutraceuticals and Food Safety, Faculty of Medicine, Thammasat University, Thailand.

### Data availability statement

Data will be made available on request.

### Declaration of interest's statement

The authors declare no conflict of interest.

### Additional information

No additional information is available for this paper.
